# Genetic characterization of a novel picorna-like virus in *Culex* spp. mosquitoes from Mozambique

**DOI:** 10.1186/s12985-018-0981-z

**Published:** 2018-04-18

**Authors:** Harindranath Cholleti, Juliette Hayer, Jose Fafetine, Mikael Berg, Anne-Lie Blomström

**Affiliations:** 1 Section of Virology, Department of Biomedical and Veterinary Public Health, Box 7028, Swedish University of Agricultural Sciences (SLU), 75007 Uppsala, Sweden; 2 SLU Global Bioinformatics Centre, Department of Animal Breeding and Genetics, Box 7023, Swedish University of Agricultural Sciences, Uppsala, Sweden; 3 Division of Molecular Diagnostics and Epidemiology, Biotechnology Center, Eduardo Mondlane University, Maputo, Mozambique

**Keywords:** *Culex*, Mosquitoes, Picorna-like virus, *Iflavirus*, RNA virus

## Abstract

**Background:**

Mosquitoes are the potential vectors for a variety of viruses that can cause diseases in the human and animal populations. Viruses in the order *Picornavirales* infect a broad range of hosts, including mosquitoes*.* In this study, we aimed to characterize a novel picorna-like virus from the *Culex* spp. of mosquitoes from the Zambezi Valley of Mozambique.

**Methods:**

The extracted RNA from mosquito pools was pre-amplified with the sequence independent single primer amplification (SISPA) method and subjected to high-throughput sequencing using the Ion Torrent platform. Reads that are classified as *Iflaviridae, Picornaviridae* and *Dicistroviridae* were assembled by CodonCode Aligner and SPAdes. Gaps between the viral contigs were sequenced by PCR. The genomic ends were analyzed by 5′ and 3′ RACE PCRs. The ORF was predicted with the NCBI ORF finder. The conserved domains were identified with ClustalW multiple sequence alignment, and a phylogenetic tree was built with MEGA. The presence of the virus in individual mosquito pools was detected by RT-PCR assay.

**Results:**

A near full-length viral genome (9740 nt) was obtained in *Culex* mosquitoes that encoded a complete ORF (3112 aa), named *Culex* picorna-like virus (CuPV-1). The predicted ORF had 38% similarity to the Hubei picorna-like virus 35. The sequence of the conserved domains, Helicase-Protease-RNA-dependent RNA polymerase, were identified by multiple sequence alignment and found to be at the 3′ end, similar to iflaviruses. Phylogenetic analysis of the putative RdRP amino acid sequences indicated that the virus clustered with members of the *Iflaviridae* family. CuPV-1 was detected in both *Culex* and *Mansonia* individual pools with low infection rates.

**Conclusions:**

The study reported a highly divergent, near full-length picorna-like virus genome from *Culex* spp. mosquitoes from Mozambique. The discovery and characterization of novel viruses in mosquitoes is an initial step, which will provide insights into mosquito-virus interaction mechanisms, genetic diversity and evolution.

**Electronic supplementary material:**

The online version of this article (10.1186/s12985-018-0981-z) contains supplementary material, which is available to authorized users.

## Background

Viruses in the order *Picornavirales* infect a variety of plants, animal hosts and insects. The order consists of five viral families, *Dicistroviridae, Iflaviridae, Marnaviridae, Picornaviridae, Secoviridae*, and an unassigned group [1]. Viruses in this order have a single-stranded, positive-sense RNA genome (+ssRNA) of approximately 9 KB in length and are non-enveloped. Most of the genomes in this order have a single open reading frame (ORF) that is flanked with a genome-linked, virus-encoded protein (VPg) at the 5′ end and a poly (A) tail at the 3′ end. The ORF encodes both structural and non-structural proteins having a conserved organization of the helicase-protease-RNA-dependent RNA polymerase (RdRp) domains [2]. The translation process is controlled by an internal ribosomal entry site (IRES) and the translated proteins are proteolytically cleaved into mature viral proteins. *Dicistroviridae* and *Iflaviridae* mainly consist of insect picorna-like viruses and are rapidly expanding families in the order *Picornavirales.* Some of these viruses are pathogenic and cause severe mortality to the host, such as deformed wing virus [3] and slow bee paralysis virus in honeybees [4].

*Culex* mosquitoes are known as potential vectors for several pathogenic viruses, such as Japanese encephalitis virus, West Nile virus and Zika virus [5–7] and are often associated with human infections and can cause mortality worldwide. In contrast, several mosquito-only viruses identified in *Culex* spp., have only been isolated from mosquitoes or mosquito cell lines [8, 9] and have no known association with vertebrates.

High-throughput sequencing (HTS) technologies have enabled the identification and genetic characterization of many novel viruses, including picorna-like viruses, from various insect hosts, such as plant hoppers [10], bean bugs [11], cotton bollworms [12], spiders [13, 14], as well as *Armigeres* and *Anopheles* mosquitoes [15, 16]. However, the picorna-like viruses in mosquitoes are poorly characterized. Previously, we have through viral metagenomics on *Culex* mosquitoes from Mozambique discovered a large number of sequences related to the *Iflaviridae* viral family [17]. In this study, we used these sequences to assemble and characterize a full-length picorna-like viral genome from the *Culex* mosquito pool using PCR and Sanger sequencing.

## Methods

### Mosquito collection, viral metagenomics and bioinformatics analysis

The mosquito collection and viral metagenomic analysis were performed in our previous study on *Culex* mosquitoes [17]. Briefly, mosquitoes were collected from Cuacua village in the Zambezi Valley of Mozambique from October–November 2014 using CDC light traps, and the genus was determined morphologically. The mosquito pools (up to 20 mosquitoes per pool) were homogenized mechanically. The total RNA was extracted with TRIzol LS reagent (Invitrogen, Life Technologies, USA) according to the manufacturer’s instructions. The samples were pre-amplified by Sequence Independent Single Primer Amplification (SISPA) and submitted to the SciLifeLab for library preparation and sequencing. The sample was sequenced on an Ion Torrent PGM sequencer with an Ion 318™ chip v2 and a max read length of 400 bp. The high-quality reads were mapped to the host genomes (*Anopheles, Aedes* and *Culex* genomes), and unmapped reads were then classified using BLASTn and BLASTx with an E-value cutoff of 1e-03. The virus-related sequences were extracted and assembled with the de novo assembler SPAdes [18].

### Sequencing of the viral genome and sequence analysis

Extracted RNA was used to synthesize cDNA using Superscript III (Invitrogen) as recommended by the supplier. First-strand synthesis was initiated with random primers. To sequence the genome gaps between the HTS assembled contigs, primers were designed, and the PCR reactions were carried out in a 25-μl reaction using the thermal profile as follows: 95 °C for 10 min; followed by 35 cycles of 95 °C for 30 s, 60–62 °C for 30 s and 72 °C for 1–1.5 min; 72 °C for 7 min. The list of the primers used in this study is enclosed as a Additional file 1. The positive PCR products were sequenced at Macrogen Europe (Macrogen Inc.) using Sanger sequencing. After the amplification and genetic characterization, Bowtie2 [19] was used to assess the genome coverage in the original HTS dataset by mapping the viral metagenomic reads back to the assembled, near full-length viral genome.

### RACE analysis of 5′ and 3′ UTRs

The extreme genomic 5′ and 3′ ends were analyzed using rapid amplification of the cDNA ends (RACE). To determine the 3′ end of the viral genome, a total of 1.2 μg of total RNA were annealed with a 0.5 μM of poly (A) specific primer (AP) in a 20 μl volume by heating at 65 °C for 5 min and chilling on ice for 2 min. First-strand cDNA was synthesized with Superscript III at 42 °C for 50 min followed by an enzyme inactivation at 70 °C for 15 min. A gene-specific forward primer (GSP-F) and universal amplification primer (UAP) were used to amplify the viral 3′ end sequences using AmpliTaq Gold DNA polymerase. For the 5′ genomic ends, 1.6 μg of total RNA and 0.5 μM of GSP-RT primer were mixed in a total volume of 20 μl, incubated at 65 °C for 5 min and chilled on ice for 2 min. First-strand synthesis was carried out by Superscript III as described previously. The RNA templates from the cDNA:RNA hybrids were degraded with 1.5 U of RNase H at 37 °C for 20 min. The excess primers from the cDNA reaction were removed using the GeneJet PCR purification kit (Thermo Fisher Scientific). The cDNA was recovered in 15 μl of elution buffer and mixed with 5 μl of 5× tailing reaction buffer, 2.5 μl of 2.5 mM dCTP and 2 μl of terminal deoxynucleotidyl transferase (Tdt) (Thermo Fisher Scientific) for 5′ end tailing at 37 °C for 15 min and then 10 min at 65 °C to inactivate the reaction. For the first round of amplification, Platinum SuperFi DNA Taq polymerase (Thermo Fisher Scientific) was used. In short, 2 μl of cDNA was used in a 25-μl reaction with 10 pmols of each GSP-reverse primer and an AUAP forward primer. The following thermo profile was used to carry out the amplification reactions: 95 °C for 2 min; 35 cycles of 95 °C for 10 s, 60–62 °C for 10 s and 68 °C for 2 min; 68 °C for 5 min and then cooling to 4 °C. The amplification products from the first round were diluted 20-fold in 1 mM EDTA. For the second round of amplification, 2 μl of diluted product and 10 pmols of GSP2- reverse primer and AUAP forward primer were used in a final volume of 25 μl, and the reaction was carried out as before. The amplified PCR products were visualized, and the bands were purified using GeneJet Gel Extraction Kit (Thermo Fisher Scientific). The purified PCR products were cloned into the pJET1.2 vector using the CloneJET PCR cloning kit (Thermo Fisher Scientific) and sequenced at Macrogen Europe.

### Analysis of the nucleotide sequence and evolutionary relationships

The open reading frame (ORF) of the viral genome was predicted using ORF finder at NCBI (https://www.ncbi.nlm.nih.gov/orffinder). The conserved domains (helicase, protease and RNA-dependent RNA polymerase (RdRP)) in the predicted ORF amino acid sequence were analyzed by multiple sequence alignment with other members of the order *Picornavirales* using ClustalW. The pairwise identity percentage matrix was generated with MegaAlign 9.0.4 (DNASTAR). To determine the phylogenetic relationships, the predicted RdRP region of different viruses belonging to the order *Picornavirales* were obtained from GenBank. ClustalW alignment of 421 amino acids (aa) corresponding to 2650–3071 aa positions were used and the phylogeny was generated using the Maximum Likelihood method based on the JTT matrix-based model with MEGA 7.0.26 software [20]. All positions containing gaps and missing data were eliminated, resulting a total of 256 aa positions of RdRP region in the final dataset. The statistical significance of the tree topologies was evaluated by 500 bootstraps. Viral sequences used in the multiple sequence alignment and phylogeny are summarized in Table 1.

**Table 1:** Summary of viruses used in the multiple sequence alignment and phylogentic analysis

Host	Virus name	Abbreviation	Genbank accession no.	Reference
Plant	Cowpea mosaic virus	CPMV	CAA25029.1	[33]
	Parsnip yellow fleck virus	PYFV	BAA03151.1	[34]
Algae	Heterosigma akashiwo RNA virus	HaRNAV	AAP97137.1	[35]
Mammals	Human poliovirus	PV	NP_041277.1	[36]
	Foot-and-mouth disease virus	FMDV	CAA25416.1	[37]
	Encephalomyocaditis virus	EMCV	NP_056777.1	[38]
	Human rhinovirus 1B	HRV	BAA00168.1	[39]
Insect	Slow bee paralysis virus	SBPV	ABS84820.1	[40]
	*Brevicoryne brassicae* virus	BrBV	YP_001285409.1	[41]
	Deformed wing virus	DWV	NP_853560.2	[23]
	*Varroa destructor* virus	VDV-1	YP_145791.1	[42]
	Ectropus obliqua picorna-like virus	EoV	AAQ64627.1	[43]
	Kakugo virus	KV	BAD06930.1	[44]
	Aphid lethal paralysis virus	ALPV	NP_733845.1	[45]
	*Perina nuda* virus	PnV	NP_277061.1	[22]
	Black queen cell virus	BQCV	NP_620564.1	[24]
	Cricket paralysis virus	CrPV	AAF80998.1	[46]
	Acute bee paralysis virus	ABPV	AAN63804.2	[47]
	Triatoma virus	TrV	AAF00472.1	[48]
	Hematobi P virus	HiPV	BAA32553.1	[49]
	Sacbrood virus	SBV	NP_049374.1	[50]
	*Plaitia stali* intestine virus	PSIV	BAA21898	[51]
	Drosophila C virus	DCV	AAC58807.1	[25]
	*Rhapalosiphum padi* virus	RhPV	NP_046155.1	[52]
	Infectious flacherie virus	IFV	ADP24157.1	[53]
	Hubei picorna-like virus 35	HplV-35	YP_009337666.1	[28]
	Hubei picorna-like virus 34	HplV-34	YP_009337152.1	[28]
	Hubei arhropod virus 1	HuAV 1	YP_009336629.1	[28]
	Lasiun niger virus 1	LniV-1	ASK12212.1	[54]
	Shuangao insect virus 8	ShiV-8	APG77945.1	[28]
	Solenopsis invicta virus 4	SINV-4	ASK12194.1	[54]
	Solenopsis invicta virus 2	SINV-2	ASK12217.1	[54]
	Hubei picorna-like virus 81	HplV-81	APG77984.1	[28]
	Lasius neglectus virus 1	LneV-1	ASK12212.1	[54]
	Armigeres iflavirus	AaIFV	LC310707	[15]
	Moku virus	MV	KU645789	[55]
Shrimp	Taura syndrome virus	TSV	ABB17263.2	[56]
Crab	Mud crab dicistrovirus	MCDV	YP_004063985.1	[57]

### Detection of the virus by RT-PCR assays

Extraction of the total RNA from *Culex* and *Mansonia* mosquito pools was performed as described above. Up to one microgram of total RNA was used for first-strand cDNA synthesis with Superscript III and random hexamers, and RT-PCR was performed with AmpliTaq Gold DNA polymerase. The PCR primers used in the assay were 8F (5’-CGACCTAGGACTTATCCAGC-3′) and 7R (5’-ACAATCTAGTGCCTCCTTCTG-3′), with an expected amplicon size of 577 bp. The PCR program was used as follows: 95 °C for 10 min, 35 cycles at 95 °C for 30 s, 60 °C for 30 s and 72 °C for 1 min, and a final extension at 72 °C for 5 min.

## Results

The viral metagenomic analysis on *Culex* spp. mosquitoes showed that the majority of the viral sequences (94.6%) were classified as the *Iflaviridae, Dicistroviridae* and *Picornaviridae* viral families [17]. The sequences were assembled into four longer contigs ranging from 578 to 2240 nt, and these contigs showed the closest similarity to Hubei picorna-like virus 35 with an amino acid identity of 37–49% (Table 2). Together, they covered approximately 60% of the Hubei picorna-like virus 35 genome (YP009337666.1). By filling the gaps between the contigs and through the RACE analysis, a nearly full-length viral genome was obtained. Unfortunately, the 3′ RACE of the viral genome was unsuccessful, while the partial 5′ UTR including complete coding sequence was obtained through a series of three successive 5′ RACE reactions. In summary, the near-full length genome, containing the entire coding region, was obtained. This sequence is 9740 nt in length and was tentatively named *Culex* picorna-like virus 1 (CuPV-1). The genome sequence was submitted to GenBank under the accession number MG833031.

**Table 2:** Best BLAST hits for the contigs assembled from *Iflaviridae*, *Dicistroviridae* and *Picornaviridae* viral reads from *Culex* spp.

Contig name	Contig length in nt	Closest relative	% Identity	Accession number
contig1	2240	Hubie picrona-like virus 35	41	YP009337666.1
contig2	1456	Hubie picrona-like virus 35	37	YP009337666.1
contig3	869	Hubie picrona-like virus 35	41	YP009337666.1
contig4	578	Hubie picrona-like virus 35	49	YP009337666.1

### Open reading frame analysis and genome organization

**Fig. 1:**
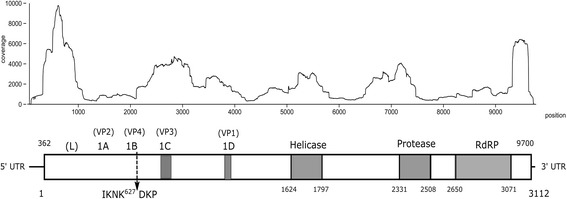
Schematic presentation of the genome of CuPV-1. The HTS reads were mapped back to the sequenced genome using Bowtie2 to display the coverage and sequence depth (upper panel). The ORF corresponds to the entire open box (lower panel). The numbers above the ORF indicate the nucleotide positions and below are the amino acid positions. In the box, the position of the putative structural proteins (1A to D) and the non-structural proteins (Leader peptide, L; Helicase; Protease; RNA-dependent RNA polymerase, RdRP) are shown. The dark areas in the ORF represent regions containing conserved sequences in the viral structural or the non-structural proteins. The dotted-arrow represents the identified cleavage site. The approximate positions of the structural and non-structural proteins were identified by the sequence similarity of other picorna-like viruses

The viral genomic sequence was found to be A/U rich (A- 32.23%, U- 31.02%, G- 21.79% and C- 14.94%). An in silico analysis of the identified nucleotide sequence of CuPV-1 showed that the genomic RNA contains a single large open reading frame (ORF) oriented from the 5′ to 3′ end. This large ORF consists of 9339 nt, encoding a 3112-amino acid protein and accounting for 95.58% of the CuPV-1 genome (Fig. 1). It has a predicted molecular mass of 352.21 kDa and theoretical isoelectric point (pI) of 5.78. The ORF was appended by a partial 5′ and 3′ UTRs which are 361 and 41 nt respectively. No large ORFs were found in the inverse orientation of the CuPV-1 genome, suggesting that the CuPV-1 genome is a positive-strand RNA virus.

### Structural proteins

Conserved domains of structural proteins were found on 5′ end of the CuPV-1 ORF. An rhv_like domain (Picornavirus capsid protein domain_like, cd00205) was identified by NCBI BLAST conserved domain suite with an E-value of 3.73e-19, at amino acids 682–876. The deduced amino acid multiple sequence analysis of the insect picorna-like viruses, including CuPV-1, revealed that CuPV-1 contains key motifs that are known to be present in the capsid proteins of picornaviruses. The conserved motifs identified on the amino acid sequences were: YXGX_8_VX_4_HX_9_F for 1C (VP3), FXRG and DDFX_7_GXP for 1D (VP1) (Additional file 2, A and B). The cleavage site for 1B/1C (NX/DXP) was also detected in CuPV-1 (Fig. 1). No conserved motifs for 1A (VP2) (NXNXFQXG) and the leader protein, the most variable region of the insect picorna-like virus genomes, were identified.

### Non-structural proteins

**Fig. 2:**
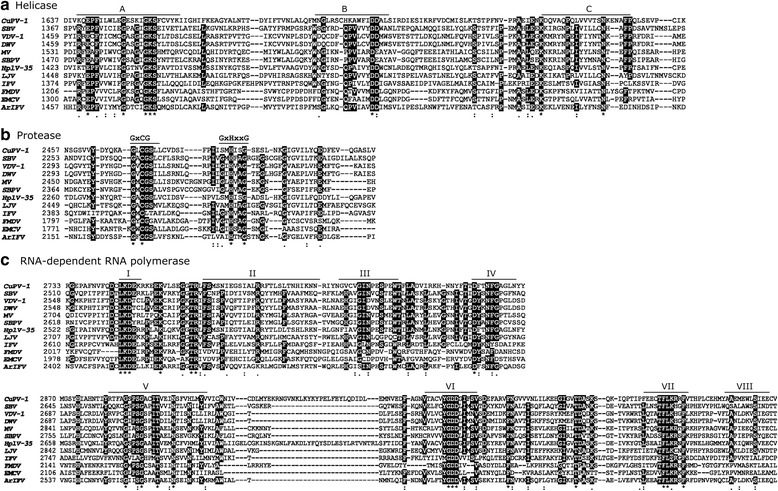
Comparison of the deduced amino acid sequences of the non-structural proteins of CuPV-1 and 11 other picorna-like viruses. **a** Alignment of the conserved regions of the putative RNA helicase region. (Full names and references of these viruses are shown in Table 1). The motifs identified by Koonin and Dolja (1993) are labeled A, B, and C. **b** Alignment of the putative protease domain of CuPV-1 with those of other viruses. **c** Alignment of the putative RNA-dependent RNA polymerase domain of CuPV-1 with those of other viruses, which are labeled I-VIII. The conserved residues are marked with asterisks (*) and residues that are identical in more than 60% of the sequences are shown in dark shades

The comparison of the CuPV-1 non-structural proteins with other picorna-like viral proteins was performed to identify the similarities and conserved regions of the putative helicase, protease and RdRP (Fig. 2).

*Helicase:* Three conserved helicase regions were recognized in the deduced amino acid sequences of the predicted CuPV-1 ORF ranging from 1624 to 1797. A conserved RNA-helicase domain (pfam00910, E-value 2.83E-11) was identified in this region. The highly conserved consensus sequence within the first domain, GXXGXGKS, was found between amino acid positions 1650–1657, although ‘K’ was substituted for ‘G’ at the 1653 amino acid position. The last two conserved domains deviated somewhat from the consensus. The highly conserved amino acids were QX_5_DD and KGX_4_SX_5_STN, while the equivalents in CuPV-1 were HX_5_DD and KDX_4_PX_5_TSN, respectively (Fig. 2a).

*Protease:* The deduced amino acid sequence of the CuPV-1’s ORF from 2331 to 2508 is similar to the protease sequence of the other picorna-like viruses [10, 21]. The conserved motif, GXCG, was found at 2469–2472, and the equivalent motif of GXHXXG, SXHXXG was found at 2485–2490. The amino acids are thought to form a catalytic triad of the protease, with the presence of H^2349^, D^2388^ and C^2471^ in this region (Fig. 2b).

*RNA-dependent RNA polymerase (RdRP):* Eight conserved domains, found between 2650 and 3071 in the deduced amino acid sequence of CuPV-1, correspond to those recognized previously, and an RNA_dep_RNAP domain (cd01699; E-value 2.52e-44) was also found in this region (Fig. 2c). This showed that the CuPV-1 RdRP belongs to a superfamily of positive-strand RNA eukaryotic viruses. The conserved or equivalent domains I-VIII in the RdRP of CuPV-1 are located in amino acids between 2743 and 3041 (Table 3). The RdRP amino acid sequence identity between CuPV-1 and closely related virus (HplV-35) was found to be 57.2%. The amino acid sequence identity matrix with RdRP regions and complete polyproteins was described in the Additional file 3.

**Table 3:** Conserved domains of RdRP amino acid sequences identified in CuPV-1

RNA-dependent RNA polymerase domains	Amino acid positions	Equivalent conserved domains in CuPV-1
I	2743–2748	DCLKDE
II	2764–2790	FSMSNIEGSIALRRFTLSLTNHIKNNR
III	2795–2807	VCVGINPESPEWT
IV	2823–2833	DFTNFGAGLNY
V	2886–2905	GSPSGACITVEINSFVHLMY
VI	2955–2965	TACVYGDDGIF
VII	3007–3019	EECTFLKRRFVTH
VIII	3027–3041	AAEMEWLSIEECVKW

### Phylogenetic relationship of CuPV-1 to other viruses in *Picornavirales* order

**Fig. 3:**
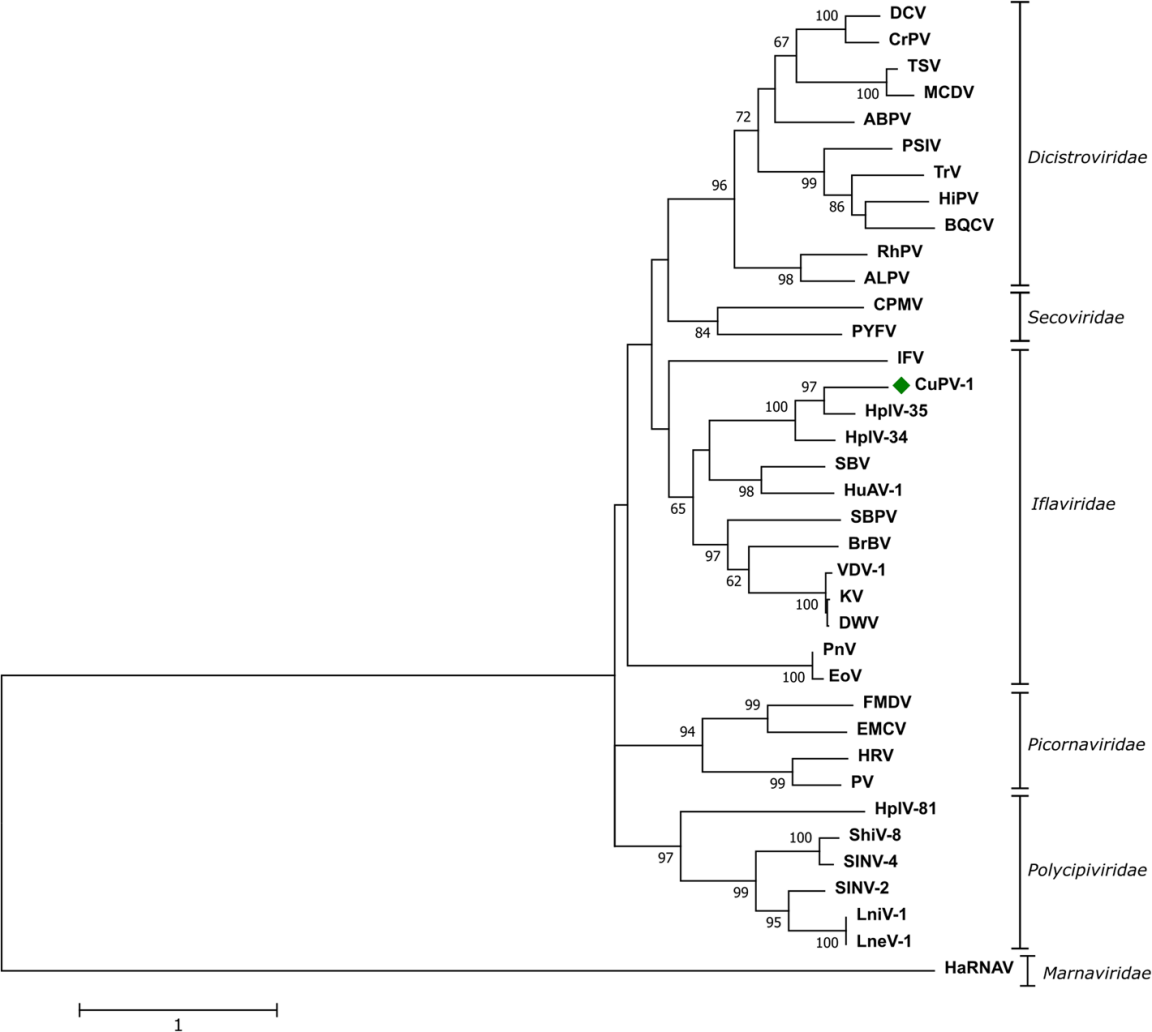
Phylogenetic analysis of the putative RdRP region. The Maximum Likelihood tree was produced, and bootstrapped at 500 replicates using MEGA7 software and involved 37 amino acid sequences, including CuPV-1. Viruses and references are listed in Table 1

To determine the phylogenetic relationship of CuPV-1 in other members of the *Picornavirales* order, phylogenetic analysis was performed using the highly conserved RdRP region including I-VIII domains. The virus formed a clade with known members of iflaviruses, such as Hubei picorna-like virus 35 and Hubei picorna-like virus 34 (unclassified picorna-like viruses), Sacbrood virus (iflavirus) and Hubei arthropod virus 1 (HuAV-1) (Fig. 3). Other clades consisted of viruses that belonged to the families, such as *Dicistroviridae, Picornaviridae, Secoviridae, Marnaviridae* and newly proposed *Polycipiviridae*. This suggests that CuPV-1 belongs to the *Iflaviridae* viral family.

### Detection of CuPV-1 in *Culex* and *Mansonia* mosquito pools

A total of 340 mosquitoes were collected in the Zambezi Valley, a central region of Mozambique. The specimens included mosquitoes of two species, *Culex* (159) and *Mansonia* (181), in 23 pools (13 *Culex* and 10 *Mansonia*). These mosquito pools were screened with RT-PCR, using primers designed for the selected CuPV-1 RdRP region. Seven pools were positive for CuPV-1, 5 from *Culex* and 2 from *Mansonia*. Each pool included 1–20 individuals and included both male and female mosquitoes (Table 4). The overall minimal infection rate (MIR), which was expressed as the number of positive pools per 1000 mosquitoes, was 0.20 (7/340), while the specific MIR for *Culex* was 0.31 (5/159) and for *Mansonia* was 0.11 (2/181). The Sanger sequencing of the positive PCR products showed that pools 9, 12, and 13 were identical to CuPV-1, however, *Culex* pools 3 and 5 showed minor sequence variation compared to both CuPV-1 (97% identity) and each other (97% identity).

**Table 4:** Mosquito pools belong to Culex and Mansonia spp. screened for CuPV-1 by RT-PCR with primers specific to RdRP region

Pool no.	Mosquito spp.	No. of mosquitoes	Positive/Negative
1	Culex	1	Negative
2	Culex	9	Negative
3	Culex	18	Positive
4	Culex	9	Negative
5	Culex	20	Positive
6	Culex	20	Negative
7	Culex	20	Negative
8	Culex	4	Negative
9	Culex	1	Positive
10	Culex	13	Negative
11	Culex	20	Negative
12	Culex	20	Positive
13	Culex	4	Positive
14	Mansonia	20	Negative
15	Mansonia	20	Negative
16	Mansonia	20	Negative
17	Mansonia	12	Positive
18	Mansonia	20	Negative
19	Mansonia	20	Negative
20	Mansonia	20	Negative
21	Mansonia	20	Negative
22	Mansonia	16	Negative
23	Mansonia	13	Positive

### Mapping of *Culex* metagenomic reads to CuPV-1 genome

The *Culex* metagenomic dataset, after the quality check, was mapped to the CuPV-1 genome using Bowtie2 to estimate the read coverage in the original data set. Among the 1,684,319 reads in the dataset, 80,636 reads (4.83% of total reads) were aligned throughout the genome with a coverage range of 9–9784 (Fig. 1), with a lower coverage towards the ends of the genome.

## Discussion

With the advances in high-throughput sequencing technologies, the number of novel viruses detected and genetically characterized has rapidly increased. Here, we report a novel and highly divergent virus sequence named *Culex* picorna-like virus 1 that was characterized from the *Culex* spp. mosquitoes by viral metagenomics and Sanger sequencing. The monopartite, monocistronic, near full-length single-stranded RNA genome (9.7 kb) was obtained. The genome encodes as single ORF coding for a 3112-amino acid polyprotein. RACE analysis was used; however, a partial 5′ UTR was obtained, which may be due to the presence of complex secondary structures of RNA, the IRES sequence and genome-linked viral proteins that interfere with cloning and sequencing [10, 22].

Multiple sequence alignments of the CuPV-1 ORF with other *Picornavirales* ORFs revealed that CuPV-1 possesses three functional motifs of helicase, protease and RNA-dependent RNA polymerases that are conserved in all members of the order *Picornavirales*. These motifs were located at the 5′ end, as observed in other picorna-like viruses [23, 24]. The non-structural protein sequences located at the 3′ end have also been found in other picorna-like viruses [24, 25]. These are involved in different functions, such as the unwinding of nucleic acids, polyprotein processing and the replication of viral genome [26, 27]. The equivalent conserved motifs for 1C and 1D were observed at the 5′ end of the ORF and showed similarities with iflavirus genome organization, suggesting that the CuPV-1 may belong to the *Iflaviridae* viral family. However, the cleavage sites of the structural and non-structural proteins of CuPV-1 need to be confirmed.

Phylogenetic analysis showed that the CuPV-1 clustered with the members of iflaviruses, further suggesting that CuPV-1 is a novel member of the *Iflaviridae* family. CuPV-1 showed closest evolutionary relationship to the unclassified picorna-like viruses Hubei picorna-like virus 35 and 34 as well as to Sacbrood virus (SBV) and HuAV-1. These viruses have all been identified in different species, *Odonata*, *Coleoptera,* honey bee and from an arthropod mix respectively, suggesting that the evolutionary relationship of different iflaviruses is not always connected to the host. This have also been seen for other iflaviruses. Novel iflaviruses from a wide range of hosts may be required to understand the evolutionary relationships of the family *Iflaviridae*. Mosquito pools that were positive for CuPV-1 in the current study suggested that CuPV-1 can infect both *Culex* and *Mansonia* mosquito species with varying infection rates. The sequence variation between the mosquito pools also indicate the presence of different CuPV-1 variants in nature. Widespread screening of mosquito species in different areas may reveal the prevalence and host range of CuPV-1.

Picorna-like viruses have been identified in a broad range of insects from the class *Insecta*. These insect viruses were classified by their genome organization, and most of them are assigned to *Iflaviridae* and *Dicistroviridae* in the order *Picornavirales*. By the recent metagenomic analyses, several viruses related to this order were also identified in the class Mammalia [28–30]. Insect picorna-like viruses are maintained in the nature by horizontal or vertical transmissions. For example, DWV can transmit from the queen honey bee to the offspring by vertical transmission and from Varroa mites to bees by horizontal transmission [3, 31]. Both, transovarial and horizontal transmissions have also been observed in SBV and *Helicoverpa armigera* iflavirus [12, 32]. A few of these viruses are pathogenic to the insect host and are also economically important, such as the infectious flacherivirus of silkworm, acute bee paralysis virus and the SBV of honeybees. However, the host range and pathogenicity of CuPV-1 needs to be further investigated. Previously, picorna-like virus (Armigeres iflavirus) has been isolated from asymptomatic *Armigeres* spp. mosquitoes [15], and, in a different study, dicistroviruses were found and believed to be members of the natural virome of *Anopheles* spp. mosquitoes [16].

## Conclusions

In the current study, the near full-length genome of a novel picorna-like virus, CuPV-1, was characterized from *Culex* spp. mosquitoes from Mozambique. The genome organization and phylogenetic analysis indicated that CuPV-1 is a novel member in the order *Picornavirales,* most likely belonging to the *Iflaviridae* family*,* and exhibit great divergence from currently known genera*.* The discovery and characterization of novel viruses in mosquitoes is an initial step that will facilitate studies on mosquito-virus interactions and pathogenesis.

## Additional files

Additional file 1: Primers used in this study. (PDF 12 kb)

Additional file 2: Conserved domains corresponding to 1C(VP3) and 1D(VP1) in CuPV-1. (PDF 489 kb)

Additional file 3: The pairwise amino acid identity matrix of CuPV-1 with other iflaviruses. (PDF 60 kb)

## Abbreviations

BLAST: Basic local alignment search tool
CuPV-1: *Culex* picorna-like virus 1
HTS: High-throughput sequencing
MIR: Minimal infection rate
ORF: Open reading frame
RACE: Rapid amplification of cDNA ends
RdRP: RNA-dependent RNA polymerase
SISPA: Sequence independent single primer amplification
