# Supplementary material for: Genetic characterization of a novel picorna-like virus in Culex spp. mosquitoes from Mozambique
Source: Virol J. 2018 Apr 18;15:71. doi: 10.1186/s12985-018-0981-z (PMC5907373; doi:10.1186/s12985-018-0981-z)
Supplement: Supplementary file 1 — Primers used in this study. (PDF 12 kb) [file 12985_2018_981_MOESM1_ESM.pdf]

Supplementary Table 1. Primers used in this study.

Primer name	Primer seqeunce 5' to 3'
5'RACE 3R	CTC TAC AGG TAT ATT CGA TCT G
5'RACE 3R nest	TCT TCT AAT CCC TGC ACC TTC
5'RACE 2R	CTC AGC TGC TCC TAC CAC AG
5'RACE 2R nest	CTC AAA CCT AGT AAA TCT CTA GG
5'RACE 1R	AAG CCT CAA CAC TCA AGT ATG
5'RACE 1R nest	CAC GTA TGC CAG GAT TTG TTC
1F	GAA CAA ATC CTG GCA TAC GTG
1R	CCCA GAA CTC ACT ATA GCT AC
2F	GGT TCT CAA CTC TGG AGT TGG
2R	AAT AAC CTG CGG TGG ATA CAC
3F	CCC AGT ATC AGG CTC TTC AAC
3R	GTC CAT TGA ACC ACT CGG AG
4F	ATG GAC ATC TTG CTA TAT GGT G
4R	TCT ATT CTC ACG CTT GGT CAC
5F	CCT GGA CTT CAT TAG CAC AAG
5R	TAG AGT CAT ATT GGA TCC ATC C
6F	ATT GTC AGA GGT ACC TTG TAT C
6R	CTC TAG AAT TAG TGG CTG TCG
7F	TGT GTT GGA GAT AGG ATC TGC
7R	ACA ATC TAG TGC CTC CTT CTG
8F	CGA CCT AGG ACT TAT CCA GC
8R	CTC CGT AAA CAC AAG CAG TAA C
9F	AAT CCC GAA TCA CCA GAA TGG
9R	TGC AGA TAT CAT AGT CCT ACT C
5' RACE AAP-FP	GGC CAC GCG TCG ACT AGT ACG GGG GGG GGG
5' RACE AUAP-FP	GGC CAC GCG TCG ACT AGT AC
3' RACE UAP	GGC CAC GCG TCG ACT AGT AC
poly (A) specific primer [illegible]	GGC CAC GCG TCG ACT AGT ACT TTT TTT TTT TTT TTT T
