# Supplementary material for: Genetic characterization of a novel picorna-like virus in Culex spp. mosquitoes from Mozambique
Source: Virol J. 2018 Apr 18;15:71. doi: 10.1186/s12985-018-0981-z (PMC5907373; doi:10.1186/s12985-018-0981-z)
Supplement: Supplementary file 2 — Conserved domains corresponding to 1C(VP3) and 1D(VP1) in CuPV-1. (PDF 489 kb) [file 12985_2018_981_MOESM2_ESM.pdf]

A. 1C (VP3)

```
                      WxGxxxxxxxxVxxxxHxxxxxxxxxP
CuPV-1    734   MHTTVAIVSSGFTNYSGGMILCGQFVKTMFHKASIEVA-IRFGR
SBV       526   QITPMEYVTGLYNFWSGPIELRFDFVSNAFHTGTVIIS-AEYNR
VDV-1     588   WFAPVGVVSSMFMQWRGSLEYRFDIIASQFHTGRLIVGYVPGLT
DWV       588   WFAPVGVVSSMFMQWRGSLEYRFDIIASQFHTGRLIVGYVPGLT
MV        597   TVPPIGVVSSLFCYWRGSIDFKFEIIATSKHTGRILVAYIPGIV
SBPV      569   YLPPISVVSSLYAYTRGSIKYKFLFGNNPRHNARLLVAYIPGIS
HplV-35   591   LSSPLGVAASFYTNYFGTMELYFQFVKTQYHKGSVEVA-IHFGR
LJV       530   VPTPLEYISSMYQFWSGPLEVRLDFVSNAFHTGSIMLS-AEFGR
IFV       573   VLPPVTYISQLFQGYTGELEYEFIPVKTAAHNFSILVAFVPFDG
FMDV      591   SNTFLAGLAQYYAQYSGTINLHFMFTGPTDAKARYMVAYAPPGM
EMCV      483   ANTFLAALSRNFAQYRGSLVYTFVFTGTAMMKGKFLIAYTPPGA
ArIFV     583   YVPPIGVVSSFFENYKGSIVYDIIAAMTDKHNIKLMFGVFPTPI
                  . :   :  :    * :            .    ..
```

B. 1D (VP1)

```
                    FxRG
CuPV-1    1040  G---MFSGSLAMTIIVDEG-
SBV       850   R---FWRGSLRYTIIIHSTD
VDV-1     1001  R---FYRGDLRFKIVFPSNV
DWV       1001  R---FYRGDLRYKIVFPSNV
MV        1040  R---FYRGGVRFRIVI-TGL
SBPV      987   R---YFRGGLRLRIVV-EGL
HplV-35   901   G---MFRGSVVYTIVLKSGI
LJV       960   R---FWRGSQRYSIISHRVT
IFV       878   R---FGRGSVILSIL--NHS
FMDV      791   RASTYYFSDLEIAV---KHE
EMCV      742   SPFVYYKCDLEVTLSPHTSG
ArIFV     994   KSCIGFKGSIILHALFPSTP
                        .
```

```
                             DDFxxxxxxGxP
CuPV-1    1182  ----KISVLLNMGDDFELGGFLGHPGFPNI
SBV       962   ----YMSVWVEAGDDFEVSNFYGPPSVKTN
VDV-1     1120  ----PVTIYYSIGDGMQFSQWVGYQPMMIL
DWV       1120  ----PVTIYYSIGDGMQFSQWVGYQPMMIL
MV        1151  ----DIAIYYTISDDCSFNVFCGFPDMVFC
SBPV      1103  ----EISVYYSIADDFSFNIFCGFPPMVYC
HplV-35   1037  ----AVDVYVNCGDDFELGGFIGHPGLANP
LJV       1073  ----TCDIFWSAGDDFDVKNFLGPPPVIAP
IFV       1021  GLQSTVTIRMGLSDDSKFHHFMGTPPVIAN
FMDV      896   ----VNELLYRMK---RAETYC-PRPLLAI
EMCV      858   ----KFTVYLRYKNK---RVFCPRPTVFFP
ArIFV     1111  ----RTVIMRSFGDDAALYLFRGFPPVVFF
                       :            :     .
```
