# Supplementary material for: Genetic characterization of a novel picorna-like virus in Culex spp. mosquitoes from Mozambique
Source: Virol J. 2018 Apr 18;15:71. doi: 10.1186/s12985-018-0981-z (PMC5907373; doi:10.1186/s12985-018-0981-z)
Supplement: Supplementary file 3 — The pairwise amino acid identity matrix of CuPV-1 with other iflaviruses. (PDF 60 kb) [file 12985_2018_981_MOESM3_ESM.pdf]

Supplementary Table 2: The pairwise amino acid identity matrix of CuPV-1 with other iflaviruses. Upper right values correspond to the % identity of RdRP I-VIII conserved domains (2743-3041 amino acid positions in CuPV-1) and lower left values correspond to the complete polyprotein.

	CuPV-1	SBV	VDV-1	DWV	IFV	HplV-35	LJV	MV	SBPV	ArIV
CuPV-1	***	33	29.5	28.8	23.7	57.2	34.9	32.4	30.8	34
SBV	20	***	45.5	46.5	34.3	37.9	56.1	44.6	41	41.3
VDV-1	14.1	18.4	***	95.8	35.3	31.5	45.8	51.9	48.4	47.8
DWV	13.8	18.6	95.2	***	35.3	32.5	47.1	51.6	48.7	47.8
IFV	14.8	16	14.7	14.8	***	24.1	32.7	33	33.7	35.3
HplV-35	38.4	24	16.5	16.9	15.9	***	36.3	34.7	32.8	32.2
LJV	20.6	33.7	17.7	18	15.7	23.3	***	43.3	40.7	44.9
MV	14.5	17.6	26.4	26.3	15.4	16.8	15.8	***	67.6	49
SBPV	15.2	16.9	26.7	26.9	15.5	17.1	16	45.5	***	44.6
ArIV	14.5	16.2	19.3	19.4	14.2	14.6	16.4	18.9	18	***
